# Syntaxin4 Interacting Protein (Synip) Binds Phosphatidylinositol (3,4,5) Triphosphate

**DOI:** 10.1371/journal.pone.0042782

**Published:** 2012-08-03

**Authors:** Tsugumichi Saito, Shuichi Okada, Atsushi Nohara, Yuko Tagaya, Aya Osaki, Shinsuke Oh-i, Hiroki Takahashi, Takafumi Tsuchiya, Koshi Hashimoto, Tetsurou Satoh, Masanobu Yamada, Jeffrey E. Pessin, Masatomo Mori

**Affiliations:** 1 Department of Medicine and Molecular science, Gunma University School of Medicine, Maebashi, Gunma, Japan; 2 Department of Medicine and Molecular Pharmacology, Albert Einstein College of Medicine, Bronx, New York, United States of America; Omaha Veterans Affairs Medical Center, United States of America

## Abstract

The insulin responsive Glut4 transport vesicles contain the v-SNARE protein Vamp2 that associate with the plasma membrane t-SNARE protein Syntaxin 4 to drive insulin-stimulated Glut4 translocation in skeletal muscle and adipocytes. The syntaxin 4 interacting protein (Synip) binds to syntaxin 4 in the basal state and dissociates in the insulin-stimulated state allowing for the subsequent binding of Vamp2 containing Glut4 vesicles and fusion with the plasma membrane. In this study, we have found that Synip binds phosphatidylinositol 3,4,5-triphosphate (PIP3), but not phosphatidylinositol 3 phosphate (PIP) or phosphatidylinositol 3,4-biphosphate (PIP2) through the Synip WW domain as deletion of this domain (Synip ΔWW) failed to bind PIP3. Over-expressed Synip ΔWW in 3T3L1 adipocytes reduced the basal levels of Glut4 at the plasma membrane with no effect on the binding to syntaxin 4 in vitro. Subcellular fractionation demonstrated that the amount of Synip ΔWW at the PM was decreased in response to insulin in 3T3L1 adipocytes whereas the amount of Synip WT increased. These data suggest that in the presence of insulin, the dissociated Synip remains anchored to the plasma membrane by binding to PIP3.

## Introduction

Insulin stimulates the tyrosine autophosphorylation of the insulin receptor (IR)-β subunit activating its tyrosine kinase activity that phosphorylates the insulin receptor substrate (IRS)-1 protein [1], [2]. Phosphorylated IRS-1 induces activation of a phosphatidylinositol (PI) 3-kinase dependent signaling cascade involving in phosphorylation of phosphatidylinositol 4,5-bisphosphate (PIP2) to phosphatidylinositol 3,4,5-trisphosphate (PIP3) [3]. PIP3 plays a central role in insulin action by inducing translocation and activation of PIP3-dependent kinase (PDK1), Akt, and the atypical protein kinase C (aPKC) [4]–[6]. This signaling pathway leads to the transport of the glucose transporter isoform 4 (Glut4) from an intracellular vesicle storage pool to the plasma membrane (PM). Recent studies of regulation of PIP3 in mice and at cellular level have shown that PIP3 plays an essential physiological role in insulin signaling and the maintenance of whole body glucose homeostasis [7], [8].

Syntaxin 4, t-SNARE protein, interacting protein (Synip) has previously been shown to function as one regulatory component of insulin stimulated Glut4 translocation in muscle and adipocytes as well as glucose-stimulated insulin secretion in beta cells [9]–[11]. Synip is composed of a PDZ domain, coiled-coil (CC) domain and WW domain. It was reported that the CC domain of Synip binds to the CC domain of syntaxin 4 [9], on the other hand the function of the PDZ and WW domains of Synip still remained unknown. Synip binds syntaxin 4 in the basal state and inhibits the interaction between syntaxin 4 and the V-SNARE vesicle associated protein-2 (Vamp2) that is present in Glut4 containing vesicles [9]. Insulin stimulation results in the Akt2-mediated phosphorylation of Synip and its dissociation from syntaxin 4 [9], [10]. These events are thought to allow Glut4 vesicle Vamp2 access to syntaxin 4 and the binding of Vamp2 to syntaxin 4 generates a fusion competent complex [12].

Glut4 translocation to and fusion with the plasma membrane requires multiple membrane lipid protein interactions. In addition to the interaction of the Synip coiled coil domain with syntaxin 4, we hypothesize that other domains of Synip might have important functions. In this regard, Synip also contains a WW domain that is 35–40 amino acids in length and contains the characteristic dual tryptophan (W) residues separated by 20–22 amino acids. WW domains are known to bind a variety of distinct peptides including motifs with core proline-rich sequences such as A/P-P-P-A/P-Y, as well as proline/arginine-containing (PR) sequences and phosphorylated serine/threonine-proline sites p(S/T)P [13]–[15]. In this report, we demonstrate that the Synip WW domain displays an atypical binding to PIP3 and that this interaction localizes Synip to the plasma membrane.

## Materials and Methods

### Materials

Dexamethasone, 3-isobutyl-methylxanthine, insulin, anti-Flag M2 antibody and Horseradish peroxidase-conjugated secondary antibodies were purchased from Sigma (St. Louis, MO). Phosphatidylinositol, Phosphatidylinositol (3) phosphate, Phosphatidylinositol (3,4) biphosphate, and Phosphatidylinositol (3,4,5) triphosphate were obtained from Echelon biosciences (Salt Lake City, UT). Calf serum and fetal bovine serum (FBS) were from Thermo Scientific (Waltham, MA), and DMEM and αMEM were from Invitrogen (Herndon, VA). The expression vector pcDNA 3.1 His was from Invitrogen (Carlsbad, CA). Anti-Glut4 (1F8) mouse monoclonal antibody and LY294002 were from Cell Signaling Technology (Danvers, MA). ECL Plus Western Blotting Detection was obtained from GE Healthcare. ProteoExtract Subcellular Proteome Extraction kit was from Calbiochem. Anti-Synip rabbit monoclonal antibody was from Epitomics, Inc. (Burlingame, CA). 3T3-L1 fibroblasts were from ATCC (Manassas, VA).

### Cell Culture

3T3-L1 fibroblasts were maintained in DMEM containing 4.5 g/liter glucose and l-glutamine supplemented with 10% calf serum, 100 U/ml penicillin, and 100 µg/ml streptomycin. Two days after confluence, cells were induced to differentiate by changing media to DMEM with 10% FBS, 0.5 mM 3-isobutyl-methylxanthine, 1 µM dexamethasone, and 1.7 µM insulin. After 4 days, the induction medium was removed and cells were maintained in DMEM with 10% FBS and insulin. Chinese Hamster Ovary (CHO) cells expressing the human insulin receptor (CHO/IR) were obtained as described previously [16]. These were maintained in αMEM containing 4.5 g/liter glucose and l-glutamine supplemented with 10% FBS.

### Plasmid Vectors

Full-length Synip inserted into pcDNA3.1 plasmid and cytoplasmic portion of syntaxin 4 inserted into pGEX 4T1 plasmid was prepared as previously described [9]. Flag-tagged Synip ΔPDZ, ΔCC, and ΔWW constructs were made by PCR and inserted into the pcDNA3.1 vector. All plasmid vectors were purified by double banding on CsCl gradients.

### Phosphatidylinositol binding by solid phase binding assay

96-well multiplates were coated with phosphatidylinositol (3 µg/ml) for one hour at room temperature, blocked with blocking buffer (50 mM Tris-HCl, 0.15 M NaCl, and 0.05% gelatin) for two hours at 37°C, and incubated with various amount of GST-fusion protein for two hours at 37°C. Bound GST fusion protein was detected using a polyclonal GST-specific antibody (0.25 µg/ml in blocking buffer) and horseradish peroxidase-conjugated secondary anti-rabbit IgG (1∶4000). Colorimetric reaction was developed using TMB substrate solution (1-Step Ultra TMB-ELISA, PIERCE). Absorbance was measured at 450 nm in a plate reader.

### Immunoblotting

Samples were separated by SDS-PAGE and electrophoretically transferred to polyvinyllidene difluoride membranes. The membranes were incubated in TBS-T (Tris Buffered Saline with 0.1% Tween20) containing 5% nonfat dry milk for two hours at room temperature and followed by incubation with monoclonal or polyclonal specific antibody as indicated in the figures and legends. Horseradish peroxidase-conjugated secondary antibodies and ECL Plus Western Blotting Detection Reagent were used for detection.

### Electroporation

This protocol was slightly modified as previously described [17]. Briefly, CHO cells were electroporated with 40 µg of plasmid under high-voltage condition (0.34 kV, 950 µF) using Gene Pulser II. For 3T3L1 adipocytes, cells were electroporated with 300 µg of plasmid under low voltage condition (0.16 kV, 950 µF). The cells were used after 48 h of recovery time. Following transfection, the cells were serum starved for four hours and stimulated with or without 100 nM insulin for 15 min at 37°C.

### GST pull down assay

*E. coli* JM109 harboring syntaxin 4 constructs was used to express proteins by induction with 0.1 mM IPTG at 24°C for 5 h. Cells were harvested, resuspended in ice-cold PBS buffer containing protease inhibitor, and then sonicated on ice. The suspensions were clarified by centrifugation and the supernatant was used for protein purification. Recombinant GST-tagged protein was purified using glutathione sepharose 4B (GE Healthcare). A GST pull-down assay was performed by mixing the CHO cell or 3T3L1 adipocytes lysate containing Flag-tagged Synip WT or Synip ΔWW stimulated with or without insulin to glutathione sepharose beads coupled with the purified GST-tagged syntaxin 4 protein. After incubation at 4°C for two hours with rotation, beads were washed with washing buffer (20 mM Hepes, pH 8.0, 0.5 mM EDTA) and the eluates were resolved by SDS-PAGE followed by immunoblot assays probing with a mouse anti-Flag antibody.

### Glut4 translocation assay

Quantification of GLUT4 translocation was determined using a colorimetric assay as previously described [10]. Briefly, 3T3L1 adipocytes were co-transfected with 400 µg of eGFP-cMyc-GLUT4 plus 600 µg of various other cDNAs as indicated in each figure. After stimulation, the cells were cooled to 4°C and incubated with a myc antibody followed by horseradish peroxidase-conjugated anti-mouse IgG antibody. The specific cell surface-bound horseradish peroxidase was then determined by mixing with the substrate, o-phenylenediamine dihydrochloride peroxidase, and absorbance was measured at 490 nm.

### Subcellular fractionation

3T3L1 adipocytes were subjected to a differential centrifugation procedure as described previously [18] using HES buffer (20 mM HEPES, 5 mM EDTA, 250 mM sucrose, pH 7.4) containing complete protease inhibitors cocktail (Roche) to yield fractions enriched in plasma membranes. All fractions were resuspended in HES buffer.

### Statistical Analysis

Data were expressed as mean ± SE from three or more experiments. Statistical analysis was performed using Student's t-test. Differences were considered statistically significant with p<0.05.

## Results and Discussion

### Synip binds PIP3 through its WW domain but not PIP or PIP2

**Figure 1:**
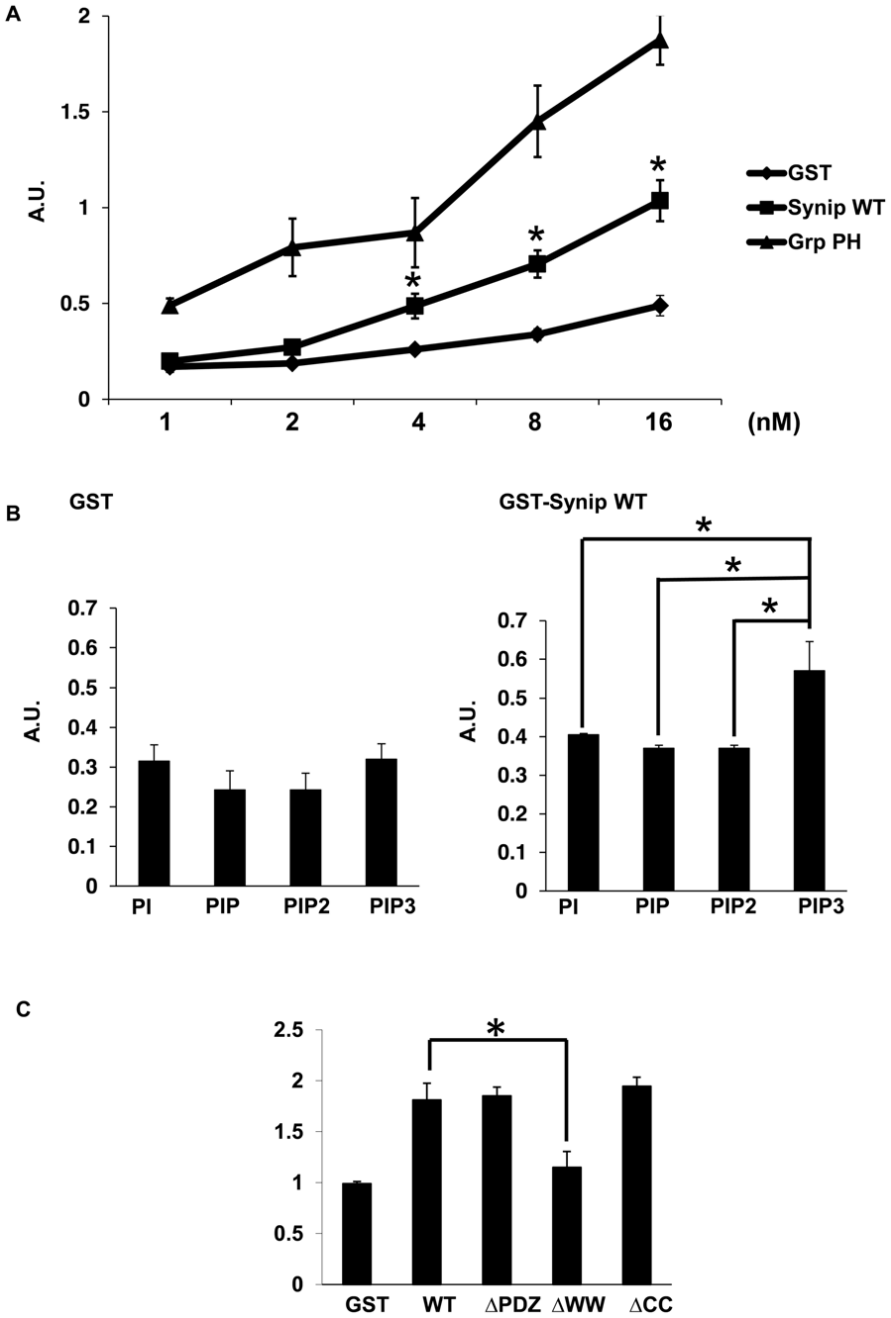
Synip binds phosphatidylinositol 3,4,5-triphosphate. (A) 96-well plates were coated with 3 µg/ml of phosphatidylinositol 3,4,5-triphosphate (PIP3) for 1 hr, followed by incubation with various amount of purified GST, GST-GRP-PH, or GST-Synip WT proteins. Bound fusion protein was detected using a polyclonal GST antibody and horseradish peroxidase-conjugated secondary anti-Rabbit IgG. Absorbance was measured at 450 nm. Data represent as means ±SE (n = 3 independent experiments), each samples run in triplicate. *, P<0.05. (B) 96-well plates were coated with 3 µg/ml of phosphatidylinositol (PI), phosphatidylinositol 3-phospahte (PIP), phosphatidylinositol 3,4-biphosphate (PIP2), or phosphatidylinositol 3,4,5-triphosphate (PIP3) and incubated with 16 nM of purified GST, or GST-Synip WT. The amount of bound GST or GST-Synip WT was determined as in (A). Data represent as means ±SE (n = 5 independent experiments), each samples run in triplicate. *, P<0.05. (C) 96-well plates were coated with 3 µg/ml of phosphatidylinositol 3,4,5-triphosphate (PIP3), followed by incubation with 16 nM of purified GST, GST-Synip WT, GST-Synip ΔPDZ, GST-Synip ΔCC, or GST-Synip ΔWW. Data represent as means ±SE (n = 3 independent experiments), each samples run in triplicate. GST, GST only; WT, GST-Synip WT; ΔPDZ, GST-Synip ΔPDZ; ΔWW, GST-Synip ΔWW; ΔCC, GST-Synip ΔCC ; *, P<0.05.

Insulin-stimulation of adipocytes rapidly increases the plasma membrane content of phosphatidylinositides, particularly PIP3 through the activation of the Class I PI-3 kinase [19]–[21]. PIP3 recruits Akt and aPKC from cytosol to plasma membrane and regulates Glut4 translocation and glucose homeostasis [7]. To determine whether Synip has the capacity to binds to PIP3, we performed lipid-protein binding assays as described in Material and Methods. As shown in Figure 1A the PH domain of GRP1 can bind to PIP3 in a dose-dependent manner compared to the control GST protein as previously reported [22]. Although Synip was not as effective as the GRP1-PH domain, there was also a specific dose-dependent binding of Synip to PIP3 compared to control GST protein.

To determine if Synip binding was selective for PIP3 or could bind other phosphatidylinositides that have also been implicated in insulin-stimulated Glut4 translocation [23], we compared the interaction of GST-Synip with PI, PIP, PIP2 and PIP3. As shown in Figure 1B, Synip displayed the strongest interaction with PIP3 among phosphatidylinositides. Deletion analysis of Synip demonstrated no significant differences in PIP3 binding between the full Synip protein or the Synip protein deleted of the PDZ domain (Synip ΔPDZ), coiled-coil domain (Synip ΔCC). However, deletion of the WW domain (Synip ΔWW) reduced PIP3 binding similar to that of GST alone (Fig. 1C). These data demonstrate that WW domain of Synip has the ability to selectively bind to PIP3.

### Over-expression of Synip WT and Synip ΔWW inhibit basal and insulin-stimulated Glut4 translocation without affecting the interaction with syntaxin 4

**Figure 2:**
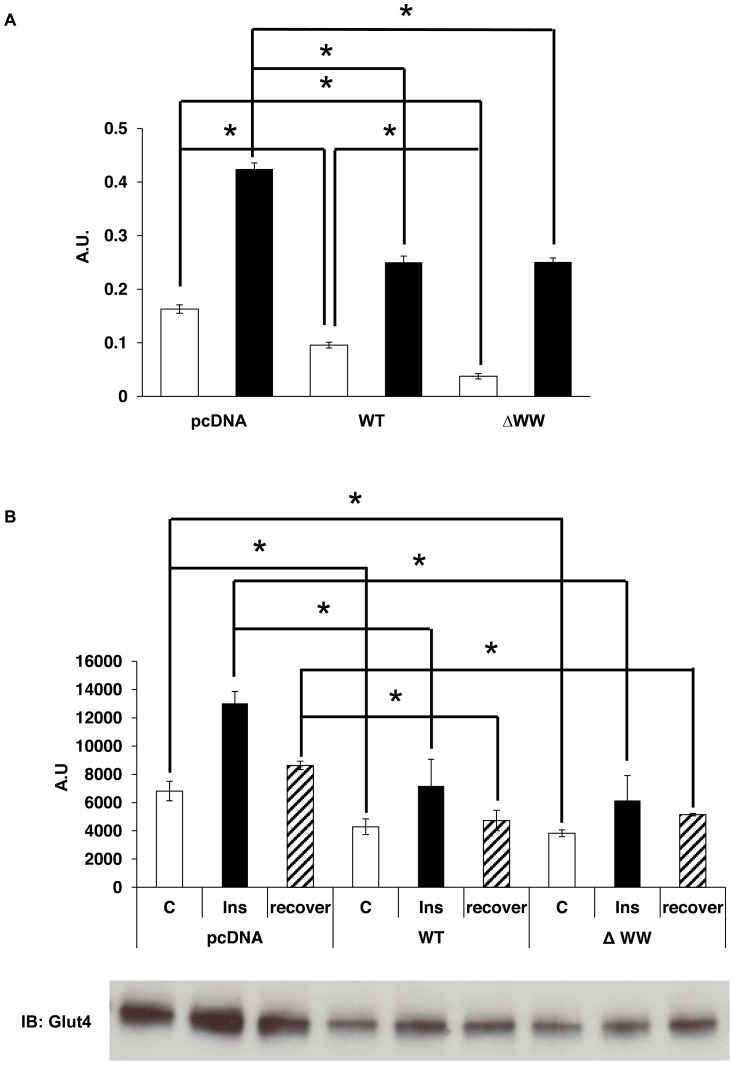
Synip delta WW domain suppressed Glut4 translocation. (A) 3T3-L1 adipocytes were transfected with Myc-Glut4-EGFP and pcDNA (vector only), Synip WT (WT), or Synip ΔWW (ΔWW) by electroporation as described in Material and Methods. After 48 h, the cells were serum starved for 2 hours and treated with or without 100 nM insulin for 10 min, fixed and incubated with anti-Myc monoclonal antibody following peroxidase-conjugated goat anti-mouse IgG antibody. The specific exofacial exposure of the Myc epitope (Myc-Glut4-GFP fusion) was detected by mixing with o-phenylenediamine dihydrochloride peroxidase and the absorbance of the mixture was read at 420 nm. Results are expressed as means ± SE (n = 3 independent experiments). Open bar, basal; closed bar, 100 nM insulin; *, P<0.05. (B) 3T3L1 adipocytes were transfected with pcDNA (vector only), Flag-Synip WT or Flag-Synip ΔWW by electroporation. After transfection, cells were serum starved for 4 hours and stimulated with or without 100 nM insulin for 15 min at 37°C. After insulin stimulation, cells were serum starved again for two hours. Then, the PM enrich fraction of cells were purified and resolved by SDS-PAGE followed by immunoblot assays probing with a mouse anti-Glut4 antibody. Evaluation of band densities was measured using NIH-Image software. Data represent as means ±SE (n = 3 independent experiments). Open bar and C, basal; closed bar and Ins, 100 nM Insulin; slash bar and recover, 2^nd^ serum starvation *, P<0.05.

To further investigate the function of WW domain of Synip, Synip WT and Synip ΔWW were over expressed in 3T3-L1 adipocytes by electroporation and Glut4 translocation assay was then monitored before and after insulin stimulation and were normalized for the relative expression levels of Synip WT and Synip ΔWW. As shown in Figure 2A, adipocytes transfected with Synip WT showed a 40% decrease in Glut4 PM exofacial myc epitope exposure both in the basal and insulin-stimulated state compared to cells expressing vector only. In contrast, expression of Synip ΔWW inhibited insulin-stimulated Glut4 PM fusion by 40% and surprisingly reduced the basal PM fused Glut4 by 70%. To confirm the suppression of Glut4 translocation to PM, 3T3L1 adipocytes transfected with pcDNA, Synip WT, or Synip ΔWW were subjected to subcellular fractionation and the relative extent of Glut4 protein present in the plasma membrane was determined (Figure 2B). In control cells, insulin increased the plasma membrane Glut4 levels and 2 h following insulin removal partially returned back to the level in unstimulated cells. In agreement with the cell surface colorimetric assay (Figure 2A), expression of Synip WT also reduced the presence of immunoblotted Glut4 in the plasma membrane fraction (Figure 2B). In this case the extent of inhibition in the basal and insulin-stimulated state was similar for both Synip WT and Synip ΔWW. The apparent Synip ΔWW induced reduction in basal PM Glut4 levels determined in Figure 2A using epitope tagged Glut4 but with no change in basal Glut4 assessed by subcellular fraction (Figure 2B) probably reflects either the nature of the two different assays or the use of over expressed versus endogenous Glut4 protein. In any case, taken together these data suggest that Synip binding to PIP3, at least for over expressed Synip, is not required for the inhibition of insulin-stimulated Glut4 translocation.

**Figure 3:**
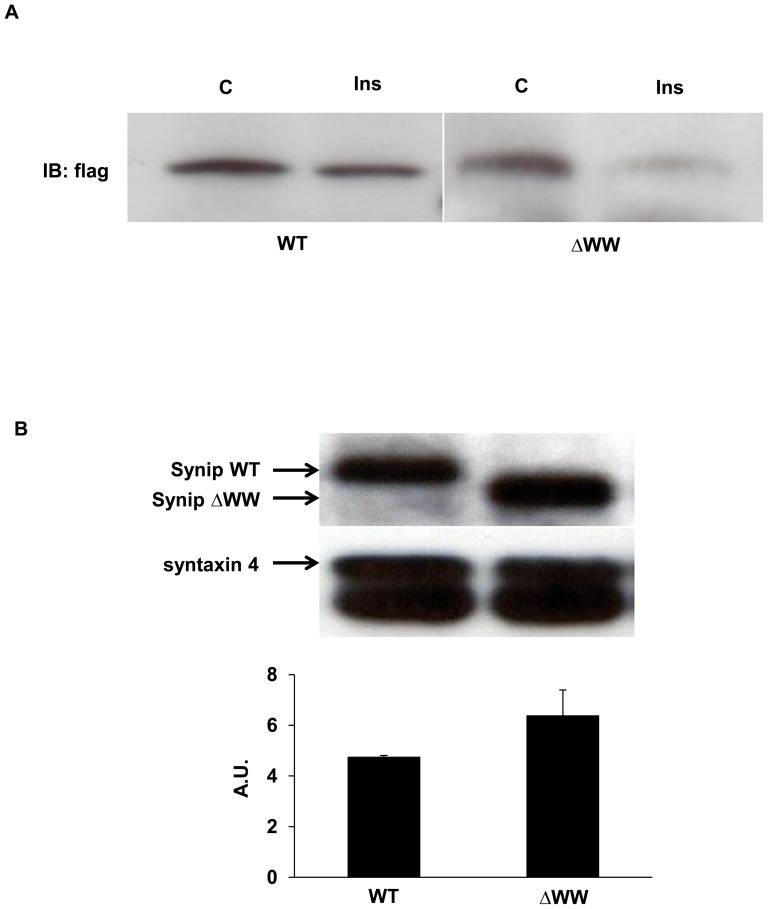
Insulin stimulation decreases the binding of Synip WT and ΔWW to syntaxin 4 in vitro. (A) Chinese Hamster Ovary (CHO) cells were transfected with Flag-Synip WT or Flag-Synip ΔWW by electroporation. After transfection, cells were serum starved for 4 hours and stimulated with or without 100 nM insulin for 15 min at 37°C. These cells were lysed with RIPA buffer and mixed glutathione sepharose beads coupled with the purified GST-syntaxin 4 protein for two hours at 4°C with rotation. After washing, elutes were resolved by SDS-PAGE followed by immunoblot assays probing with a mouse anti-Flag antibody. (B) 3T3L1 adipocytes were transfected with Flag-Synip WT or Flag-Synip ΔWW by electroporation. After transfection, cells were serum starved for 4 hours and lysed with RIPA buffer and mixed glutathione sepharose beads coupled with the purified GST-syntaxin 4 protein for two hours at 4°C with rotation. After washing, elutes were resolved by SDS-PAGE followed by immunoblot assays probing with a mouse anti-Flag antibody or anti-syntaxin 4 antibody as a control. Results are expressed as means ± SE (n = 3 independent experiments).

Since insulin reduces the binding of Synip to syntaxin 4, we examined the binding of Synip WT and Synip ΔWW to GST-syntaxin 4 in cell extracts. As shown in Figure 3A, both Synip WT and Synip ΔWW associated with syntaxin 4 from control cell extracts and this interaction was reduced in cell extracts from insulin-stimulated cells. To estimate the amount of Synip WT and Synip ΔWW binding to syntaxin 4 in the basal state, cell extracts from transfected 3T3L1 adipocytes were incubated with GST-syntaxin 4. As shown in Figure 3B, Synip ΔWW binding to syntaxin 4 was not statistically different than the binding of Synip WT. These results indicated that Synip WT and Synip ΔWW bind endogenous syntaxin 4 and based upon previous findings, result in blocking syntaxin 4-Vamp2 interaction that suppresses plasma membrane Glut4 localization in basal and insulin stimulated condition.

### Synip WW domain is important for the localization at plasma membrane through PIP3 after insulin stimulation

**Figure 4:**
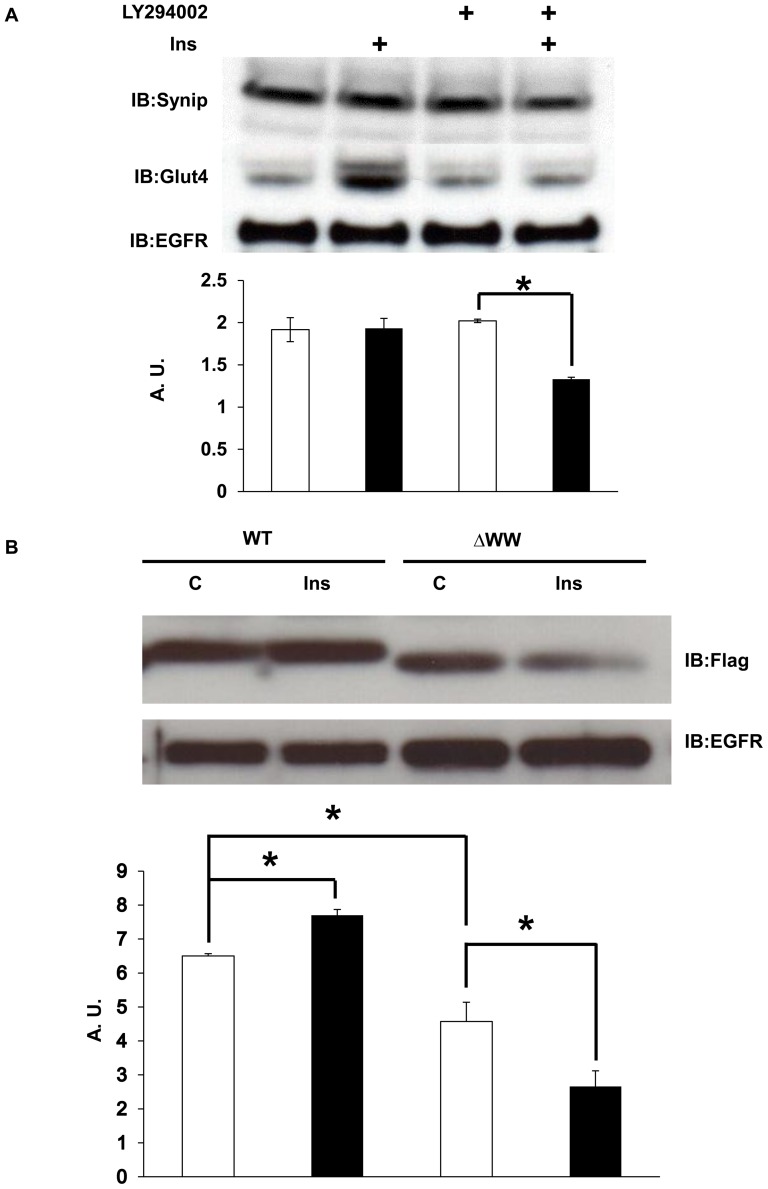
Synip membrane association following insulin stimulation is dependent on the WW domain. (A) Differentiated 3T3L1 adipocytes were serum starved for six hours and treated with 100 nM insulin for 15 min in the presence or absence of 25 µM LY294002 for 30 min. The cells were then fractionated using different centrifugation as described in Material and Methods. The fractions enriched PM (10 µg) were resolved on a 10% SDS-PAGE and subjected to Western blotting using anti-Synip antibody, anti-Glut4 antibody or EGF receptor antibody as a loading control. Bottom panel, quantification of plasma membrane Synip protein. (B) 3T3L1 adipocytes were transfected with Flag-Synip WT or Flag-Synip ΔWW by electroporation. After twenty-four hours, the cells were serum starved for six hours and treated with or without 100 nM insulin for 15 min. The cells were then fractionated using ProteoExtract Subcellular Proteome Extraction kit according to the suppliers' protocols. The membrane fractions (10 µg) were resolved on a 10% SDS-PAGE and subjected to Western blotting using anti-mouse Flag antibody or EGF receptor antibody as a loading control. Evaluation of band densities was measured using NIH-Image software. Data are expressed as means ± SE (n = 3 independent experiments). Open bar and C, basal; closed bar and Ins, 100 nM Insulin.

In the pancreatic beta cell line βHC-9, Synip localizes at PM in both the basal and glucose-stimulated state [11]. As Synip dissociates from syntaxin 4 in the stimulated state another domain must be responsible for maintaining membrane localization. In this regard, the WW domain of Pin1 was previously found to be necessary for Pin1 intracellular localization [24] and as shown in Figure 1 the Synip WW domain can bind to PIP3, a PM localized phosphoinositide. Next we investigated whether Synip localize at PM through PIP3 in 3T3L1 adipocytes. Differentiated 3T3L1 adipocytes pretreated with or without LY 294002 were stimulated with 100 nM insulin and then isolated PM rich fraction. Consistent with our previous finding in βHC-9, the amount of Synip present in isolated PM by subcellular fraction was not significantly different between basal or insulin-stimulated 3T3L1 adipocytes (Fig. 4A), despite the ability of insulin to dissociate Synip from syntaxin 4 (Fig. 3A). The plasma membrane localization of Synip was dependent on PIP3 as the PI3 kinase inhibitor blocked insulin-stimulated Glut4 translocation and reduced Synip localization at the plasma membrane.

To determine whether WW domain of Synip is needed for membrane localization of Synip, we analyzed the membrane association of flag-Synip WT or flag-Synip ΔWW transfected into 3T3L1 adipocytes. As shown in Figure 4B, Synip WT localized to the membrane fraction in both the basal and insulin-stimulated states, and in multiple experiments there was in fact a statistically significant increase in membrane association following insulin stimulation. In contrast, the basal amount of Synip ΔWW localized to the PM was significantly less compared to that of Synip WT. Moreover, insulin stimulation resulted in a marked decrease in the PM associated of the Synip ΔWW mutant.

Taken together, the data presented in this study demonstrates the Synip WW domain has the capacity to bind to PIP3. Although WW domains are typically thought to function as a protein-protein interaction module bind to proline-rich motifs, phosphothreonine and phosphoserine residues [25], WW domains are highly prolific and a relative low affinity for PIP3 compared to PH domains (ie. Grp1-PH domain) in retrospect it is not that surprising. In the case of Synip, the coiled-coil domain appears to be necessary for the basal membrane association through its interaction with syntaxin 4, that also serves to inhibit Vamp2 binding and Glut4 transport vesicle fusion with the PM. However, following insulin stimulation that dissociates Synip from syntaxin 4, Synip remains membrane bound through the engagement of the WW domain with the insulin-stimulated production of PIP3. This in turn will maintain Synip in close proximity to syntaxin 4 such that when the insulin signal is terminated and PIP3 levels decrease, Synip can readily re-associate with syntaxin 4 preventing any further fusion of Glut4 with the PM.
